# Policy Framework and Barriers in Antimicrobial Consumption Monitoring at the National Level: A Qualitative Study from Pakistan

**DOI:** 10.3390/antibiotics15010089

**Published:** 2026-01-15

**Authors:** Beenish Ihsan, Shahid Muhammad Iqbal, Mohammed Aufy, QurratulAin Jamil

**Affiliations:** 1 Department of Pharmacy Practice, Faculty of Pharmacy, The Islamia University of Bahawalpur, Bahawalpur 63100, Pakistan; beenishihsan27@gmail.com; 2 Department of Pharmacology, Faculty of Pharmacy, The Islamia University of Bahawalpur, Bahawalpur 63100, Pakistan; 3 Michael Sars Center, University of Bergen, N-5006 Bergen, Norway; 4 Division of Pharmacology and Toxicology, University of Vienna, UZA II, Josef-Holaubek-Platz 2, A-1090 Vienna, Austria

**Keywords:** antimicrobial consumption, AWaRe, policy framework, one health approach, antimicrobial footprint, antimicrobial resistance, national action plan

## Abstract

**Objectives:** The study aims to assess the strategies used to estimate antimicrobial consumption (AMC) and the barriers encountered in data collection. It also addresses the perception about AMC based on the World Health Organization (WHO) definition. **Methodology:** The qualitative study adhered to the standard consolidated criteria for reporting qualitative studies (COREQ) guidelines. It involved stakeholders from diverse sectors, i.e., regulatory bodies, the pharmaceutical industry, international health organizations, policy experts, medical professionals, veterinary doctors, and academia (nursing, medicine, and pharmacy). A total of 37 in-depth interviews were conducted using a semi-structured interview schema. The interviews were recorded and transcribed verbatim. Codes were generated afterward and organized into themes. **Results:** Data analysis yielded five themes consisting of (i) Perception about Antimicrobial Consumption, AWaRe (Access, Watch, Reserve) classification and related terms, (ii) Antimicrobial Consumption: Policy Design, (iii) Data management and record keeping for the Estimation of Antimicrobial Consumption, (iv) Levels of Estimation for Antimicrobial consumption and Organizations, and (v) Challenges and suggested solutions in estimation of AMC: One health approach is the way forward. **Conclusions:** The study concluded that AMC and AMR are two sides of the same coin. The solution to AMR and excessive AMC is to re-evaluate the policy and implement legislation strictly. Efforts focused on irrational prescribing and unsupervised OTC sales of antimicrobials. This will help in reducing the consumption of broad-spectrum antimicrobials.

## 1. Introduction

Antimicrobial resistance (AMR) has appeared as one of the most serious public health challenges of the 21st century [1]. Antimicrobials are consumed as both curative and preventative drugs for infectious diseases [2]. Two primary contributors to the development of resistance are inadequate and excessive use of antibiotics [3]. The global threat posed by AMR to mankind is escalating as existing antimicrobial medicines are becoming less effective due to the emergence of resistant strains of microorganisms [1].

The consequences of drug resistance are poor patient outcomes, the spread of infections, and increased healthcare costs [4]. AMR is a natural phenomenon, but inappropriate antimicrobial use accelerates its emergence [5]. The World Health Organization (WHO) has acknowledged the significance of a more effective and consistent global response to contain AMR. The WHO’s Global Strategy for Antimicrobial Resistance Containment was published in 2001, and it outlines a series of interventions designed to combat the emergence and spread of antimicrobial-resistant bacteria [6,7].

Antimicrobial use (AMU) and antimicrobial consumption (AMC) are two different yet critical interrelated terms. Both sets of data are complementary and serve specific purposes in understanding antimicrobial patterns [8]. Monitoring antimicrobial consumption and use can provide critical insights into usage patterns and identify inappropriate use. One key objective of the Global Action Plan on AMR is to gather evidence regarding AMR and antibiotic use through surveillance [9].

Adoption of the One Health approach facilitates cross-sectoral collaboration, linking human, animal, and environmental health sectors under a unified framework to address the severity of AMR effectively [10]. In Pakistan, the Drug Regulatory Authority of Pakistan (DRAP) is a major stakeholder that monitors the AMC and works with both national and international organizations to improve data management and surveillance related to AMC and AMU.

This qualitative study, conducted in Pakistan, explores legislation, regulatory frameworks, and policy implementation related to AMC in a One-Health context. This study aimed to determine the perceptions, challenges, barriers, limitations, and policy gaps of different stakeholders. It also discusses what impedes raising awareness of the consequences of inappropriate antibiotic use and the reasons for the inability to control over the counter (OTC) sales of antimicrobials. This study also explains why understanding and policy on consumption data is crucial if we want to develop a surveillance system for antimicrobials.

## 2. Results

**Figure 1:**
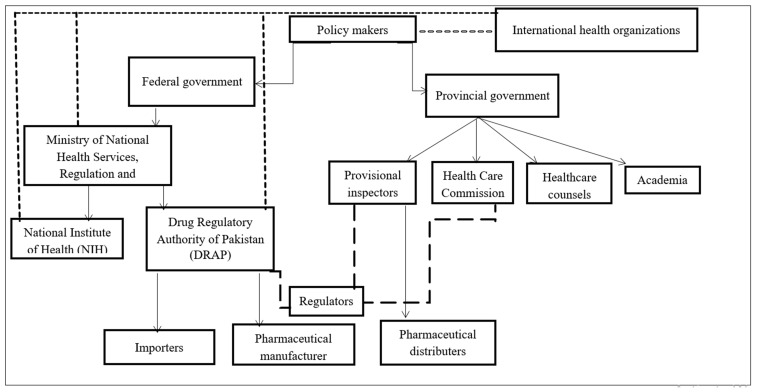
Tiers of antimicrobial Consumption and key stakeholders. 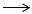 indicates a direct connection and impact of stakeholders with respect to AMC, 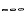 works in mutual interest (indicates aligned interest however not directly affected), 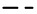 indicates regulators from different disciplines however connected (involved in AMU and AMC).

A total of 39 people showed interest, and 37 participants were interviewed. A total of five themes evolved. Demographic details, including the organizations, ages, professions, and experience of the participants, are presented in Table 1 and Table 2. Figure 1 provides an overview of the stakeholders, linking them to AMC.

### 2.1. Theme I: Perception About Antimicrobial Consumption, AWaRe Classification, and Related Terms

This theme included understanding and awareness of basic terminology among relevant stakeholders. It was observed that stakeholders directly involved in preparing or implementing the National Action Plan (NAP) for antimicrobial resistance had better knowledge than other participants.

#### 2.1.1. Perception About Antimicrobial Consumption and Antimicrobial Use

The terms AMC and AMU were presented to experts to assess their understanding and perception of these terms. Participants working in organizations directly involved in AMC, such as the DRAP and international organizations, were able to differentiate between the two terms according to the WHO’s definition. However, pharmaceutical manufacturers, importers, distributors, and other stakeholders believed that AMC and AMU are the same. Of 37 participants, only 7 were able to differentiate AMC according to the WHO definition.

An officer from DRAP explained the difference between the two terms.

“There is a narrow difference. Antimicrobial consumption is used as an overall term for how many antimicrobials are imported in Pakistan, while utilization is the number of medicines or antimicrobials used in a hospital.”

(Federal regulator-I)

Another respondent stated,

“In my point of view, AMU and AMC are different. AMU is a rational use of antimicrobials, while AMC is the use of antimicrobials in your country. AMC is the consumption of antimicrobials in all aspects, whether it is legal or illegal.”

(International health organization-I)

Most of the respondents believed that both terms are the same and can be used interchangeably.

“I believe antimicrobial consumption and use are more or less the same, i.e., the total amount of antibiotics manufactured or imported in a country.”

(Federal regulator-II)

“The use is the same as consumption; I don’t see any difference between the two.”

(Manufacturer-III)

#### 2.1.2. Antimicrobial Resistance

Respondents highlighted it as a significant problem of the present day. AMR needs serious recognition at all levels, from policymakers to healthcare professionals to the public.

“AMR is the major problem. We further elaborate on the terms’ consumption’ and ‘utilization’ under AMR. Irrational use and self-medication are the primary drivers of this issue. Many superbugs are changing, which we knew during treatment.”

(Pharmaceutical policy and practice expert-I)

All stakeholders were aware of AMR and understood its severity.

#### 2.1.3. AWaRe Classification

The majority of respondents who were familiar with the AWaRe classification of antimicrobials were directly involved in implementing the NAP (13 out of 37). All the other respondents had little to no knowledge about it. However, they emphasize the need to raise awareness among healthcare professionals. According to the expert, healthcare professionals lack understanding of the essential medicines list (EML) and AWaRe.

“WHO has an essential medicine list, which is used as it is without any amendment, and is approved by DRAP.”

(Federal regulator-IV)

Another respondent stated

“I think that every country should adopt not just the WHO classification but its own form of classification, which should be assessed according to their needs, because resistant patterns are different. However, the WHO classification is helpful for the country as well as any region, for example, developing countries like Pakistan, which have limited resources.”

(Federal regulator-III)

Experts from government and international health organizations strongly believe that healthcare professionals and new trainees must be aware of the rational use of antimicrobials and related terminology. They should be educated during their graduation, and training sessions should be held. The National Institute of Health (NIH), a major stakeholder in AMR, should take the lead in collaboration with state and local health departments.

“Some physicians have no idea about AWaRe, and I don’t blame them for that, as they are not provided with any awareness about it. They have not been trained in any way, and this should be the responsibility of the provincial departments and the federal government.”

(International health organization-I)

Only four respondents were familiar with terms such as daily defined doses (DDDs) and its use in estimating AMC. Similarly, only four respondents knew the prescribed daily dose (PDD) and its use in calculating AMU. Respondents who were direct stakeholders and linked directly to AMC were familiar with DDD and PDD. Most respondents from academia and the manufacturing industry were unaware. Thus, it is essential to train all stakeholders to calculate DDDs and PDDs to estimate AMC and AMU better, respectively.

### 2.2. Theme II: Antimicrobial Consumption: Policy Design

The theme “Antimicrobial Consumption: Policy Design” covers import, manufacturing, distribution, and sales, as well as the rules governing them.

#### 2.2.1. Rules for Import and Manufacturing of Antimicrobials

The respondents were asked about existing legislation for the import, manufacturing, and sales of antimicrobials. The legislation regarding imports, manufacturing, and sales is similar to that for other medicines. All products imported by manufacturers are registered, and the DRAP maintains an import record. The manufacturers are bound by rules to submit quarterly consumption reports of raw materials of all drugs, including antimicrobials, but this is not being followed.

“Sub rule 6 of rule 30 of Licensing, Registration and Advertising Rules under the Drug Act 1976, states that all the manufacturers that use API, will submit their consumption record quarterly on form 7 (L, R &A), and unfortunately, there is no implementation of that rule.”

(Provincial regulator-III)

“Drug registering, licensing, and advertising are rules framed under the drug act 1976. It states that each manufacturing unit of medicines whose products are registered must provide import, export, and production records every 3 months. It is not implemented in the true spirit.”

(Provincial regulator-V)

DRAP maintains records and permissions for the import of all therapeutic goods, including antimicrobials.

“DRAP has data on all anti-microbial drugs imported into Pakistan. The import and export section within the quality assurance and lab testing (QA&LT) division regulates the import and export of antimicrobial raw materials and finished products. DRAP is the authority primarily responsible for approving the import of health commodities by the public and private sectors, importers, individual organizations, and public institutions (e.g., hospitals, donor agencies, and research institutions). It issues all import permits for any medicine imported into the country. However, the consumption record of those antimicrobials is not always kept up-to-date.”

(Federal regulator-IV)

A respondent from the industry stated

“We obtain an import license before importing the antibiotics and a manufacturing license before formulating it. Similarly, the distribution and sale of drugs are regulated and conducted in accordance with the relevant regulations after obtaining the relevant license. However, we are not strictly asked to submit quarterly consumption.”

(Manufacturer-II)

Another respondent stated

“We import antimicrobials to meet the demand of the market, and it is beneficial from a business point of view. Seasonal trends of specific antibiotics also exist. In winter, the sales and demand of antibiotics increase. We don’t have any import restrictions regarding the quantity of antibiotics”

(Manufacturer-I)

A manufacturer stated that the situation and market are challenging for them now. They follow all procedures and import the medicines legally. Mainly, quarterly reports are submitted to ensure the amount of the drug manufactured.

“We follow rules, and we register drugs and import as per policy and submit the consumption reports quarterly. Inspectors visit to assess the quality of materials and ensure SOPs are followed during manufacturing. If quality is not maintained, the industry is fined and sealed by the inspector depending on the offense”

(Manufacturer-III)

The manufacturer from another industry revealed,

“Though we are bound to submit a quarterly consumption report, we usually don’t. Annual reports are submitted occasionally or when we need to import or export another consignment of drugs; we are also asked to report the consumption of the old import. In this way, the next consignment is cleared. However, unlike controlled drugs, there is no restriction on the amount of antibiotics we can import if we are registered for the specific drug.”

An officer from DRAP commented that it is easier to estimate AMC in the human sector; however, it is more difficult in the animal sector.

“Some antimicrobials used in the animal sector are imported as growth promoters, so these are imported without approval of DRAP. Moreover, the same antimicrobial is administered in various animals, and to calculate DDDs, we have to divide it by different animal weights for each type of animal and it is often not done.”

(Federal regulator III)

Legislation regarding the import, export, manufacture, and sale of antimicrobials exists in Pakistan. But the legislation must be implemented in the true spirit. Respondents also stated that the amount of antimicrobials to be imported is not restricted like narcotic and psychotropic substances.

#### 2.2.2. Distribution and Sales: Legislation in Different Provinces of Pakistan

Rules regarding the sale and dispensing of antibiotics without a prescription apply in all provinces of Pakistan, as these are not registered as OTC products. However, there are loopholes: many drugs are antibiotics but are not listed in the relevant schedules. We need to revise and amend the policy and laws. Moreover, there is no specific section on antimicrobials in the legislation. However, the existing laws, [provincial drug sales rules (schedule D)], restrict the sales of drugs without a prescription, which are not implemented in the true spirit, leading to excessive consumption and use of antibiotics. The respondents commented that these laws were not updated and lacked many aspects.

“Seven set rules regarding sales are implemented in our country, including all four provinces (Punjab, Sindh, Baluchistan, Khyber Pakhtunkhwa (KPK), Islamabad Capital Territory, AJK, and Gilgit Baltistan. There are two types of schedules in these rules. The important one is Schedule D of drug rules, which is implemented in Punjab, Islamabad, and KPK. There is a list of medicines in the schedule that may be sold only with a prescription, and you must maintain records of their sale. If these schedules are checked strictly, you can see that these schedules are not updated, so you can’t achieve results even after their 100% implementation.”

(Federal regulator-I)

It is strictly forbidden to dispense the antimicrobials, and it should be clearly mentioned on the medicine that it should not be sold without a prescription.

“There are the Drugs (Labeling and Packing) rules 1986, stating the medicines that fall in the schedules (schedule D) should be labeled ‘to be sold on the prescription of a registered medical practitioner (RMP) only’. We are deficient in both the implementation and legislative aspects.”

(Provincial regulator-III)

Another regulator commented,

“In Pakistan, mostly antibiotics are sold without a prescription. After a struggle of 3–4 years, we have controlled the sale of psychotropic and controlled drugs. Now, we can start working on antimicrobials to be sold only with a prescription.”

(Federal regulator-IV)

All provinces have their own regulatory bodies. Respondents stated that they collaborate with the federal DRAP. Mostly, the provincial authorities have their own roles to fill.

“All the provinces have their regulatory bodies. There are no specific sales rules for the antibiotics in the provinces. The EML and schedules are not up-to-date, and many antibiotics are not included in the relevant schedules.”

(Federal regulator-I)

“Both human and animal drugs are referred to as therapeutic drugs, and the distribution and consumption among the human and animal industries have the same laws.”

(Provincial regulator-V)

Antimicrobials are not listed in the prescription-only medicine list.

“The rules that exist are not followed because of many issues. Many antibiotics are not part of the list of ‘sold on prescription only’. The rules and lists need amendments; we are working on it.”

(Provincial regulator-V)

All stakeholders agreed that there is a dire need to implement laws and that they should be amended as needed, on a priority basis.

### 2.3. Theme III: Data Management and Record Keeping for the Estimation of Antimicrobial Consumption

The estimation of consumption in animals and humans, and the management of the import and export of antimicrobials, are discussed under this theme.

#### 2.3.1. Estimation of Consumption: Grounds for Human and Animal Consumption

Respondents noted that, to date, consumption is not calculated at a higher level, and no proper published records exist at the national level. Since DRAP is the major stakeholder for AMC, they are working hard to maintain the records. Respondents from international organizations stated that the role of DRAP should be highlighted, and it should play its part responsibly for AMC.

“DRAP should play this role, they are working on this aspect to be more helpful as they are the major stakeholder.”

(International health organization-IV)

In humans, consumption can be estimated at different levels, from the consumer level to the high import level. A regulator highlighted the multidisciplinary role of DRAP and the stakeholders’ role in antimicrobial data management. Unfortunately, DRAP does not have complete records of manufacturing or distribution; the data available is incomplete and inadequately documented.

This is the need of the hour to document the data so we can identify the problems. It will help identify loopholes and highlight deficient areas.

“DRAP has a role in the registration of data. We have the import and export data here. The manufacturers have manufacturing and distribution records for finished products. Distributors have the data for further distribution of antimicrobials to retailers and community Pharmacies.”

(Federal regulator-II)

The respondent stated that many private organizations are working on collecting data. They have their own methods of collecting data.

“IQVIA takes data from distributions and a few private setups, as our government setups don’t have resources, training, and manpower to collect the data systematically”

(International health organization-II)

International organizations are also working to analyze available data to estimate consumption. At the individual level, few studies have been conducted to understand the situation. A representative from an international organization stated his opinion on estimating antimicrobials using import or trade data to get a broader picture.

“There are many methods; the one we have used till now is to document the international trade, meaning data from customs, import in animal and human health.”

(Federal regulator-IV)

A representative from the regulatory authority stated,

“We have import and export data of antimicrobials, but sometimes it is difficult to segregate human and animal data. A manufacturer can have a human and veterinary antimicrobial section under the same license, though the sections are separate. So, if they imported antimicrobials, it is difficult to segregate whether the antimicrobial was imported for human medicine or for veterinary medicine”

(Federal regulator-V)

Another representative stated,

“Import and export data are documented in kilogram/units. In both the animal and human sectors, generic drugs are imported. Active ingredients are imported in kilograms. We haven’t reached the level to maintain data by route of administration. We compile the import and export data and keep it. Recently, we started segregating the data we receive by access watch and reserve groups. We don’t have further segregation.”

(Federal regulator-III)

Respondents from the health sector stated that point prevalence surveys are conducted to estimate antimicrobial use. They emphasized the need for point prevalence surveys in both the animal and human sectors. The respondent from a health organization discussed the status of consumption work in Pakistan, and the biggest challenge is validating data, as utilization and consumption patterns sometimes differ, leading to gaps.

“After obtaining data, the next step is validation of that data, e.g., if ciprofloxacin is imported 40 tons, its manufacture and consumption don’t need to be according to the import; there will be a difference in the pattern of how it is utilized. We need to calculate the import data and report it, and simultaneously begin estimating the utilization pattern. For utilization, the process is not easy. We have to approach this at different levels within the government sector, such as what kinds of procurement executive district officer (EDOs), district health officers (DHOs), medical superintendent (MS), Tertiary care, and other offices can do. For now, it is not possible to obtain data from all sectors and every hospital, so we can start by selecting a few locations to collect data, and we have started with KPK. We haven’t started in other places till now because of a feasibility issue.”

(International health organization-III)

#### 2.3.2. In Animals (as Feed Grade and Growth Promoters)

Respondents discussed how measuring consumption should be based on different grounds for humans and animals. Many medicines are not imported as antimicrobials but as growth promoters. In animals, the AMC and AMU vary depending on the animals, i.e., poultry, livestock, and fisheries, as the weight of the animals varies. In the animal sector, an international organization, in collaboration with academia, has worked on estimating consumption at the farm level on a smaller scale to provide a picture of the situation across different provinces.

“There are different ways of monitoring consumption. In animal health, there is a concept of using a raw material, a pure material, or an active pharmaceutical ingredient (API). Few companies in Pakistan import antibiotics as nutritional products, calling them feed grade, and DRAP is not involved in it, as they are not directly labeled as antibiotics. I don’t know how consumption data is interpreted and evaluated internationally. Still, in Pakistan, I believe it cannot happen accurately; we cannot estimate consumption on similar grounds as humans, as in the animal sector, we have finished products very rarely.”

(International health organization-IV)

Respondents working in the veterinary sector share knowledge and experience on how to estimate data on animal consumption at the farm level.

“Only a sophisticated farm keeps the record of use. We struggled a lot and tried many ways, like installing trash cans and asking them to throw medicine-related trash into them. Every brand has almost 4,5 combinations of antimicrobials in one dosage form of medicine. In such a situation, calculating AMU becomes complicated. Those who maintain the record are not doing so because they have concerns about AMR; they have to keep a ledger of the amounts used from a business point of view.”

(Academia animal health-I)

The respondents stated that, since no proper record-keeping system existed, national and international organizations are working hard to develop one. The system developed might help to keep and maintain the records. All respondents agree that a proper system for the segregation of antimicrobials imported for human and animal use is needed.

### 2.4. Theme IV: Levels of Estimation for Antimicrobial Consumption and Organizations

The level of estimation for AMC and AMU, as identified by the study’s stakeholders, is presented in Table 3.

#### 2.4.1. Responsibilities of Different Organizations for Controlling Antimicrobial Consumption and Resistance as per the National Action Plan

Respondents stated that antimicrobial resistance is due to the irrational use and overprescribing.

“Major focal point for AMR in Pakistan is NIH, which is working in collaboration with health departments, regulatory bodies, federal (DRAP), provincial), hospitals, and academia for controlling AMR. DRAP deals with the AMC component (the NIH’s role is collaborative), i.e., the import, manufacturing, distribution, and sale of antimicrobials. The mandate of international health organizations working in Pakistan is to collaborate with national and provisional bodies and assist them, such as providing training sessions, and other matters.”

(Pharmaceutical policy and practice expert-I)

The responsibilities of major stakeholders on AMC and AMR, based on the respondents’ views, are detailed in Table 4. All the other stakeholders, like academia, the animal sector, the environment, agriculture, and hospitals, are directly or indirectly working with the mentioned stakeholders. Hospitals work independently to develop AMU guidelines or follow international guidelines.

#### 2.4.2. Software for Data Collection and Data Arrangement

The respondent stated that DRAP was working on software named Pakistan Integrated Regulatory Information Management System (PIRIMS) in collaboration with other organizations, like USAID-funded organizations (promoting quality of medicine (PQM) to keep track of the drug manufacturing and licensing). The project has been launched and is in its initial stages; DRAP is working to implement an effective system to reduce the workload. If the AMU and AMC are interlinked, they can see a wider picture; estimating either alone can give a clear picture of the situation. The respondent explained that software is essential because the data will be digitized, enabling productive use and saving time and energy.

“We have launched PIRISMS, for the licensing of pharmaceutical units. This will improve accountability and transparency. Once we shift it online, it will reduce the workload and give accurate trends of import and export of drugs.”

(Federal regulator I)

This is the first step after they achieve the target and shift the licensing online. They plan to adopt a multi-department approach to combine data on the import and export of antimicrobials and other medicines with consumption trends.

“Our vision is that when we get the ultimate trend for the anti-microbial consumption data, we want to link it with the AMR pattern. With a linked resistance pattern, we can determine whether specific antimicrobials are required. This information will help medical professionals and other stakeholders.”

(Federal regulator-II)

Relevant stakeholders stated that developing proper software is a need of the hour. It will reduce effort and easily segregate antimicrobials used for human and animal consumption.

### 2.5. Theme V: Challenges and Suggested Solutions in Estimation of AMC: One Health Approach Is the Way Forward

#### 2.5.1. Challenges

The respondents discussed the challenges to estimating the consumption. Lack of a reporting system is one of the significant problems. The existing data-collection system has many loopholes and was not digitized previously. Now, DRAP is working to collect data electronically, but it still faces many challenges. The data is not submitted regularly, leading to missing data at both the manufacturer and hospital levels. Hence, the available data contains many discrepancies. The respondents said that the resources to estimate the consumption are very scarce. The reasons and challenges faced for AMC estimation are stated in Table 5.

Respondent from a federal organization summarized the issue and stated their opinion. Collecting data from provincial governments can be an issue because they don’t share data on time or omit important information. Lack of will is a significant reason, as he believed that where there is will, there is a way.

“Government organizations don’t have a proper system. Even in the organization, there is a conflict of opinion. Sometimes, availability and accessing data are issues, indicating a lack of proper systems and coordination among departments. Ideally, every province should have a system that compiles and reports data monthly, quarterly, and annually to the director general of health of the province; they then report it to the national organization, for example, in case of AMR to NIH & for AMC to DRAP. The national organizations should consolidate the data, generate an annual report on the country’s situation on that matter, and share it with the WHO for the GLASS report. In our country, data is not compiled regularly or on time, leading to missing data and broken links. We do not have institutionalization; we receive the data but lack the resources or methods to validate it. Sometimes we obtained data that is flawed to the point that carbapenems are not used on poultry, but the data will support their use, showing a major error.”

(Federal regulator-1)

A respondent stated that the provincial and federal governments are not well coordinated. The data they receive is sometimes incomplete. Since they have no proper online system and software, they have to rely on them.

“Data reliability is a major issue, and gaps exist. Survey-based data has its own limitations. As we don’t have a system available, it’s better to have something than nothing. Some private organizations collect data, and the ways adopted to collect data lead to a question mark.”

(Federal regulator-III)

Lack of training was stated as one of the reasons.

“International organizations gave training. I believe they were not very fruitful. We need to understand that training should have a practical aspect rather than just bookish, repetitive knowledge. They should improve the training content. At our organizational level, we also offer interdepartmental training. But not much was about the AMC aspect.”

(Federal regulator-II)

A representative of an international health organization blamed the federal government for being weak and failing to play its part.

“For AMC, weak institutions in terms of federal regulatory authority, poor communication between provincial and federal authorities, and a lack of digitalization are the main problems. If customs can digitalize it through a single-window operation, why can’t the federal organization primarily responsible for AMC do the same? The transfer of officers responsible for AMC to other departments is another challenge. This makes it difficult to design a proper training.”

(International health organization-I)

Another representative who has experience working in the government sector and is now a representative of an international organization, upon asking about the situation where organizations blame each other, said

“Objectively speaking, lack of trust, miscommunication, or no communication at all are the primary reasons that lead to many problems. The government has power, and international organizations have funding. If they can sit and give credit where it’s due, it can help resolve many issues.”

When asked by a government respondent, they offer a different perspective. An expert working in provincial government stated budgetary constraints as one of the significant reasons. It restricts the government from hiring employees, leading to a lack of manpower, and overburdened staff end up doing extra work after completing the job they were hired for. This leads to delays.

“This is a developing country, money is an issue, and people work overtime to earn money. If you have money, you can hire more people & have a well-developed system.”

(Provincial government-I)

A respondent stated that the lack of legislative implementation is also due to limited resources and a shortage of staff, which leads to many issues.

“I agree, it is the weakness, and I believe it is a system failure, as we are unable to stop or restrict OTC sales of antibiotics. Realistically speaking, we are a developing country, and people are poor. To avoid physicians’ charges, they self-medicate or prefer the quacks, a cheaper solution. They visit qualified doctors or professionals only when the situation is worse, as the areas are remote and travelling costs a lot. We cannot change the situation overnight. It will take time, and I believe strict implementation of laws is the only solution”

(Provincial government-II)

The respondent stated that surveillance plans are needed to collect epidemiological data, given the scarcity of existing data, and to generate statistics.

“The human health surveillance plan raises objections; it is too microbiology-oriented, and the epidemiology part is missing.”

(Federal regulator-V)

A respondent from an international organization stated that, in addition to the use of antibiotics in the animal and human sectors, the excessive use of antibiotics and pesticides in agriculture is a significant concern and a challenge.

“We need to check the use of pesticides and antibiotics in agriculture. People lack awareness of the issue’s sensitivity. Those pesticides enter the soil, contaminating the environment and the food consumed by humans and animals. It is a vicious cycle.”

(Agriculture-I)

#### 2.5.2. Suggested Solutions

Respondents stated that AMR and AMC estimation are issues that can only be addressed through communication and collaborative efforts among different sectors. A summary of the proposed solutions is given in Table 6. Various steps to improve the consumption and use are necessary to address this issue. The expert stated that, regardless of the situation, the only thing he believes can improve it is strict enforcement of the law and a will to work for change.

“The best solution is to change our behavior; we should change ourselves rather than trying to change others. The government also needs to implement strict rules on self-medication with antibiotics. Strict implementation of laws and reviewing the old rules to close the loopholes are the solution.”

(Academia pharmacy-II)

The Pharmaceutical Policy and Practice Expert (PPPE) expert and federal government representative believed that generic prescribing is essential to control irrational prescribing. He talked about the steps the government has taken on this issue.

“As you know, DRAP wrote the generic letter, which states that no physician is allowed to write the antimicrobials with brand names and only use the generic name so that misuse of antibiotics can be prevented. Even physicians cannot differentiate between antibiotic brands, and they suggest two different brands without realizing that both are the same. DRAP is pressing hard on the provinces to sit together with the secretary of health, the Healthcare Commission, and other relevant authorities of the provinces and agree on a common point not to give any antibiotics without a prescription and dispense only in the presence of a pharmacist.”

(Pharmaceutical policy and practice expert-I)

The federal government respondent provided insight into plans.

“In the past, we invited 2 to 3 people from every province to the headquarters, but now we have planned to go to each province to train and guide them. I am now responsible for organizing the AMC and AMS awareness campaign, and I have written letters to all provinces to maintain records of antimicrobial drug data. We aim to educate the doctors, nurses, pharmacists, pathologists, and medical lab technicians.”

(Federal regulator-III)

The development of user-friendly software to digitize data will play a significant role in the success of our efforts to estimate the AMC. Many respondents highlighted the lack of proper software. Now the PIRISMS is working, but it is in the initial stage.

“Development of user-friendly software that could be used while offline as well is mandatory. As Pakistan is a developing country, and all the provinces or areas do not have a smooth internet connection.”

(Provincial regulator-III)

The respondent from academia stated that our students should be made aware of the guidelines during their studies.

“Educate the medical students and pharmacy students about AMR, international guidelines, AWaRe classification may be one of the solutions.”

(Academia medicine-1)

A representative from the Healthcare Commission stated,

“Regulation of sales of antimicrobials is the responsibility of DRAP or provincial drug inspectors. Our work is to make sure the clinics have qualified doctors. Ensuring the presence of a pharmacist at pharmacies is the responsibility of drug inspectors. However, we are trying our best.”

(Healthcare Commission-I)

Mapping the antibiotic supply chain and maintaining records in both the human and animal sectors will help track the antibiotic footprint. International and national health organizations are working together to get a snapshot of the situation. This will help policymakers and prescribers.

“We did a few surveys whose results are under process of compilation. We will share our results with veterinary drug prescribers and provide recommendations. We forward our recommendations only after we have results based on evidence.”

(International organization-II)

The respondents suggested that surveillance programs for AMR, AMU, and AMS should be launched not only in the human and animal sectors but also in the agriculture and environmental sectors.

“Currently, we have launched the AMS programme for veterinary drugs. Surveillance programmes and AMS should be implemented in both the human and animal sectors”

(International health organization-I)

A representative of an international health organization stated that the solution to all our problems is adopting a One-Health approach. This will help all stakeholders stay on the same page and collaborate to address the situation. The centralized system, where the data is collected and shared with relevant departments on a need basis, is essential. In line with the National Action Plan, steps are being taken to map the data. Individually, organizations are working in their capacity; all we need is to build trust among stakeholders and work together to get a clear picture.

## 3. Discussion

AMR is a global and One-Health concern that impacts the environment, healthcare, and food production systems, as well as people, animals, and plants. This implies that it should also be addressed throughout these sectors, with a wide range of stakeholders, and at all levels. To attain a high degree of protection for human health, antimicrobial consumption (AMC) optimization and increased public knowledge of antimicrobials and AMR are important [11,12].

This study provides a detailed perspective on AMC and AMU, knowledge of estimation methods, reasons for excessive antimicrobial consumption, barriers to estimating AMC, and loopholes in the policy framework. The fourth objective of the NAP for antimicrobial resistance of Pakistan is regarding the prevention of irrational antibiotic prescribing and irrational combination [13]. Adopting a One-Health approach and implementing antimicrobial stewardship programs were suggested solutions to address AMR. These steps will pave the path for smooth estimation of AMC. Our respondents highlighted the gaps, which included (i) lack of resources and regulatory capacity, (ii) lack of high-level governmental support, (iii) lack of involvement and collaboration among relevant stakeholders, and (iv) lack of engagement and advocacy by professional associations and society groups to create awareness.

AWaRe classification is a helpful tool developed by WHO in 2017 for maintaining data on antibiotic consumption, setting goals, and assessing the results of stewardship programs to maximize antibiotic consumption and reduce antimicrobial resistance [14,15]. Similarly, to the findings of a study conducted in Jordan, which revealed that only a few respondents were aware of the AWaRe classification of antimicrobials, whereas most of them were unable to differentiate and describe the significance of the classification [16]. Stakeholders’ perceptions of the differences between AMC and AMU vary. The stakeholders directly involved or working on AMR in any capacity were familiar with the difference proposed by WHO [8].

The findings of this study discussed the existing policy framework for the estimation and regulation of sales of antimicrobials according to the Drug Act 1976 [17]. The laws exist, but OTC sales of antimicrobials are not strictly regulated, leading to excessive use and, in turn, excessive AMR. These results were similar to a study conducted in Romania, where chain pharmacies did not dispense OTC antimicrobials, whereas small businesses dispensed them without a prescription, leading to irrational use [18]. Another study from Brazil shows a decrease in the consumption of antimicrobials after policy implementation [19]. Many studies from Pakistan highlighted the issue of dispensing of antimicrobials without rational prescription [20], and overuse of antimicrobials is making Pakistan a country in the list of Low and Middle Income Countries (LMICs) with the highest consumption of antimicrobials [21].

A study discussed that DRAP issued notifications to ensure generic prescribing. Similarly, findings of this study revealed efforts of regulatory authorities to highlight the role of generic prescribing that may help in avoiding the duplication of antimicrobials in a prescription [22,23].

A review of the regulatory framework shows that India has a robust legislative framework that prohibits the OTC sale, import, and manufacture of antimicrobials without a license issued by the authorities. No medicines can be marketed without a quality check [24]. Our study revealed similar findings, stating the presence of a regulatory framework. The findings highlighted that the quality of antimicrobials is frequently checked, and all measures are taken to ensure quality. The manufacturers and importers are required to submit a quarterly report [17]. However, our study found that a lack of manpower and resources is a reason for the poor implementation of the law requiring quarterly antimicrobial consumption reports. The study revealed that the policy is lenient. Trained professionals and mass campaigns are essential to heighten public awareness of the issue. A similar scenario is highlighted in review articles [25,26].

The study findings reveal that the consumption can be estimated at different levels. Import data represents a bigger picture of the situation. The estimation can then be narrowed down to manufacturers and distributors to compare data. This will help to ensure transparency and remove discrepancies. PPS at the hospital level will estimate antimicrobial utilization for a specific disease at the hospital or ward level. These findings suggest representatives working on AMC and AMR know the guidelines of the WHO [8]. In animals, antimicrobials are given with food, leading to higher consumption without the animal’s knowledge. Antibiotics used in agriculture, fisheries, and the animal sector as growth promoters are alarmingly high. That is an indirect source of antimicrobial consumed in humans. These findings are in line with a study conducted in Malawi, where antimicrobials were given in food and vitamin mixes, leading to higher consumption in livestock [27].

The respondents believed that AMC and AMU are interlinked, and a clear picture of the situation is essential to tackle AMR, requiring extensive research through surveillance programs, as suggested by other experts [28].

The findings reveal that no proper system has yet been developed to address the AMC situation. Lack of software and properly trained manpower leads to missing data links. Currently, the primary focus in our country is to evaluate antimicrobial resistance in microorganisms. The respondents state that they cannot emphasize its importance more. Still, rational prescribing and a strict policy to control OTC sales of antimicrobials are equally important, as they both contribute to AMR. A One-Health approach focusing also on the animal sector, agriculture, the environment, and antimicrobial stewardship programs, along with a well-developed surveillance system, was suggested as a possible solution. Moreover, proper infrastructure was also required. Similar findings were reported in other studies [29].

In Pakistan, the primary stakeholder for collecting and managing antimicrobial data is DRAP, the federal regulatory authority. National organizations (DRAP and NIH), with support from international organizations such as FAO, WHO, and the Fleming Fund, aim to address existing data gaps and strengthen policy frameworks through stakeholder collaboration. This study thoroughly evaluated the state of the policy framework for AMC in Pakistan; however, it has several limitations. Although a broad range of stakeholders was involved, specific crucial stakeholders, including retail and community pharmacists, were excluded, as the focus was on regulatory and manufacturing aspects and on national-level data estimation to get a broader picture. The study also focuses on the need for a One-Health approach to map the antibiotic footprint and on the involvement of stakeholders from academia, the veterinary industry, the environment, and agriculture. Potential response bias may have occurred due to the use of both in-person and telephone interviews; however, detailed information packs and assurances of confidentiality helped ensure openness and reliability of responses.

## 4. Materials and Methods

The qualitative study, employing in-depth interviews, was conducted from January 2024 to July 2024, adhering to the Consolidated Criteria for Reporting Qualitative Studies (COREQ) guidelines by adopting a purposive convenience sampling technique [30]. Key participants from the human and animal health sectors were selected for their roles in policymaking and implementation. Participants included national and provisional policymakers, representatives from regulatory bodies and drug authorities, the pharmaceutical industry, healthcare commissions, international organizations in Pakistan, and academia (Table 1 and Table 2 in Section 2).

To conduct this study, invitations to interviews were sent via email and phone. If no response was received after two reminders, the invitation was deemed declined. A total of 45 participants were contacted, of whom 39 responded to the invitation. Two participants withdrew later due to recording concerns. A total of 37 interviews were conducted: 29 in the human health sector and 7 in the animal health sector. One respondent advocated for both the human and animal health sectors.

The inclusion criteria included: (a) participants having working experience of more than 2 years; (b) participants having direct influence on either policy or AMC; the participants represented a large group of people involved in AMC directly or indirectly in Pakistan.

The interviews ranged in duration from 25 to 80 min. The mean interview time was 38.22 ± 15.47 min.

### 4.1. Interview Schema Development

The schema was developed with an extensive literature review (Supplementary File S1) by BI and reviewed by QAJ and SMI. The schema focuses on antimicrobial consumption (AMC), legislation for monitoring of AMC, and challenges to estimating AMC. An extensive literature search was done to familiarize and to remain unbiased. The interview schema was pilot tested through interviews with one pharmacist from DRAP and one physician from the WHO engaged in AMC and AMR. Interviews were recorded, transcribed verbatim, and validated.

### 4.2. Data Collection

Interviews were conducted face to face, by telephone, and via the Zoom meeting app by BI (a female researcher and PhD candidate) with 5 years of experience in qualitative research. A semi-structured interview scheme was used to conduct the interviews. The participants were briefed on the research topic, assured of confidentiality, and informed that participation in the interviews was voluntary and that they could walk away at any time. All sessions took place privately, and permission was obtained before recording. The interviews were recorded on a mobile phone and via the Zoom video conferencing application, and the tapes were saved on a password-protected computer. Important field notes were taken during or immediately after the interviews to better understand the interviews. No repeat interviews were conducted. No financial incentives were offered. All the interviews were conducted in English. Every audio recording was transcribed verbatim by author BI. Transcribed files were shared with participants for verification; lack of response after two reminders implied approval.

### 4.3. Data Analysis

Thematic analysis was performed manually employing the technique outlined by Braun and Clark [31]. Transcripts were initially read in depth to make notes and record essential themes and codes. A fundamental coding framework outlining the subthemes, categories, codes, and quotes was then created. In the final round, the research team refined the codes even further. To protect anonymity, identifiable information was removed from all research records. To enable comparison of excerpts from the same person, each transcript was coded with an interview number and the relevant sector. We adopted a deductive approach as our coding guide, allowing codes that did not fit the framework to emerge inductively as new topics. BI coded all the interviews in the first round, followed by the second round, during which the authors BI and QAJ merged similar codes. The research team discussed the final themes and then categorized them and their sub-themes based on the results. To ensure consistency between findings and the data presented, triangulation principles were used [32], in which, under the same theme, perspectives of at least three stakeholders with distinct differences and common ground were presented to provide a complete picture of the situation. Thematic saturation criteria [32] were used to identify “rich information” within each topic, and we concluded that no new themes, subthemes, or codes emerged from the data and further interviews were not conducted.

## 5. Conclusions

The study concluded that enhancing the capabilities of already trained task forces by developing a user-friendly software system capable of operating offline and maintaining import records is essential for effective antimicrobial data management. Additional manpower and strict enforcement of legislation are required to ensure reliable data collection, particularly in remote or hard-to-reach areas. It also concluded that excessive antimicrobial consumption primarily results from irrational use, which contributes to antimicrobial resistance and treatment failure. Tracking the antimicrobial footprint is vital to protecting human, animal, and environmental health. Addressing this issue demands policy re-evaluation, strict enforcement of rules, and effective control of irrational prescribing and OTC sales. Strengthening AMS programs can promote rational prescribing and dispensing. Enhancing the education of physicians and pharmacists, both in academia and practice, is essential to ensure appropriate antimicrobial use. Adopting the One-Health approach and engaging all stakeholders offers the most sustainable path to reduce misuse and control AMR.

## Figures and Tables

**Table 1:** Characteristics of stakeholders.

Type of Institution	Sector				Total
	Human Health			Animal Health	
Academia	Medicine	Nursing	Pharmacy		
	2	2	2	2	8
Federal government	8				8
Provincial government	5				5
Pharmaceutical policy and practice expert	1				1
International organizations	3			2	5
Manufacturers/Importers	5			2	7
Healthcare Commission	3			--	3
Total	31			6	37 *

* 7 of the stakeholders were PhDs (doctoral degrees) in the respective fields. It indicates the highest level of expertise of respondents.

**Table 2:** Qualification and experience of stakeholders.

Details	Number of Participants
Profession	
Doctor (MBBS)	8
Veterinary doctor	4
Pharmacist	19
Nurse	2
Distributor/Manufacturer	4
Gender	
Male	29
Female	8
Age	
<40	15
40–60	18
>60	4
Experience (years)	
<5	3
5–10	10
10–20	14
20+	10

**Table 3:** Levels of estimation of AMC and AMU (discussed based on WHO definition of AMC and AMU).

Levels of Estimation of AMC	Levels of Estimation of AMU
Customs data	Retail pharmacies
Import data from DRAP	Hospitals (private and public sector)
Manufacturers	Private clinics
Distributors	

**Table 4:** Identified stakeholders and responsibilities of different stakeholders of AMC and AMR in Pakistan, from an antimicrobial consumption perspective.

Organization	Major Stakeholder	Responsibilities
DRAP	AMC	▪ Registration and licensing of drugs ▪ Maintaining a record of import and export of medicines ▪ Granting permission for advertising medicines ▪ Policy making
NIH	AMR	▪ Implementation of the national action plan on AMR ▪ Reference laboratory ▪ Microbiological aspect ▪ Surveillance programs ▪ Antimicrobial Stewardship implementation
International health organization (not a major stakeholder, but assists)	AMR, AMC, AMU	▪ Assisting (in epidemiology surveys, surveillance, immunization, ▪ Provide funding ▪ Work with the national organization according to international standards and provide training and assistance ▪ Help in improving policies ▪ Helps promote evidence-based practice ▪ Helps identify gaps
Provincial government		Distribution and sale of drugs

**Table 5:** Key challenges and reasons listed by stakeholders highlighted for collection, maintaining, and estimation of data on AMC (bold captions highlight categories in this subtheme) (numbers indicate total responses).

**Limited resources for the estimation of AMC** ▪ Lack of human resources (30) ▪ Lack of specialized people working in this domain (18) ▪ Budget issues (25)
**Too much focus on the microbial resistance aspect instead of AMC (2)**
**Less communication between provincial and federal authorities (4)**
**Lack of training sessions** ▪ Lack of proper and professional training sessions (19)
**Lack of IT software** Lack of digitalization (provincial) (11)
**Legislative Issues** (Lack of legislation and its implementation) ▪ Lack of implementation of dispensing laws in both sectors (29) ▪ Lack of implementation policy for the OTC sale of antimicrobials at the national or provincial level (30) ▪ No revision of old rules (new antibiotics are not updated) (6)

**Table 6:** Proposed solutions offered by stakeholders for estimating AMC and controlling AMR (bold captions highlight categories in this subtheme) (Numbers indicate total responses).

**Improving and implementing existing legislations** ▪ Identify gaps in legislation (17) ▪ Strict rules against the sale of antibiotics without a prescription (33) ▪ Regulation of sales (17)
**Capacity building programs** ▪ Hire more professionals (13) ▪ Ensure the presence of a qualified professional (17) ▪ Allocate the seats and budget in respective provinces (6) ▪ A pharmacist should be present in every pharmacy (21) ▪ Awareness and training seminars for HCPs (17) ▪ Educate the medical students and pharmacy students about AMC, AMU, AMR, and AMS (3)
**Development of user-friendly software that could be used while offline** ▪ Centralized system (10)
**Mapping of the antibiotic supply chain and keeping records in both the human and animal sectors** ▪ Antibiotic footprint (3) ▪ Implementation of AMS in Hospitals (18) ▪ Surveillance programs (18)
**One Health Approach** ▪ Developing the proper infrastructure for implementing the One Health approach (6)
**Mass Awareness campaigns (27)**

## Data Availability

The original contributions presented in this study are included in the article/Supplementary Materials. Further inquiries can be directed to the corresponding authors.

## Supplementary Materials

The following supporting information can be downloaded at https://www.mdpi.com/article/10.3390/antibiotics15010089/s1, File S1: COREQ (COnsolidated criteria for REporting Qualitative research) Checklist. Ref. [32] is cited in the Supplementary Materials.

## Abbreviations

The following abbreviations are used in this manuscript:

AMC	Antimicrobial Consumption
AMS	Antimicrobial Stewardship
AMR	Antimicrobial Resistance
API	Active Pharmaceutical Ingredient
AWaRe	Access, Watch, Reserve
COREQ	Consolidated Criteria for Reporting Qualitative Research
DRAP	Drug Regulatory Authority of Pakistan
FAO	Food and Agriculture Organization
GLASS	Global Antimicrobial Resistance and Use Surveillance System
KPK	Khyber Pakhtunkhwa
NIH	National Institute of Health
OTC	Over the Counter
PIRIMS	Pakistan Integrated Regulatory Information Management System
PQM	Promoting the quality of medicine
RMP	Register Medical Practitioner
USAID	United States Agency for International Development
WHO	World Health Organization
LMICs	Low and Middle Income Countries
